# Fully automated segmentation of substantia nigra toward longitudinal analysis of Parkinson’s disease

**DOI:** 10.1007/s11548-025-03451-9

**Published:** 2025-10-06

**Authors:** Tao Hu, Hayato Itoh, Masahiro Oda, Shinji Saiki, Koji Kamagata, Kei-ichi Ishikawa, Wataru Sako, Nobutaka Hattori, Shigeki Aoki, Kensaku Mori

**Affiliations:** 1 Graduate School of Informatics, Nagoya University, Furo-cho, Chikusa-ku, Nagoya, 464-8601 Japan; 2 Department of Applied Mathematics, Faculty of Science, Fukuoka University, Nanakuma 8-19-1, Jonan-ku, Fukuoka, 814-0180 Japan; 3 Information Technology Center, Nagoya University, Furo-cho, Chikusa-ku, Nagoya, 464-8601 Japan; 4 Department of Neurology, Institute of Medicine, University of Tsukuba, Tennodai 1-1-1, Tsukuba, 305-8575 Japan; 5 Department of Radiology, Juntendo University Graduate School of Medicine, Hongo 2-1-1, Bunkyo-ku, Tokyo, 113-8421 Japan; 6 Department of Neurology, Juntendo University School of Medicine, Hongo 2-1-1, Bunkyo-ku, Tokyo, 113-8421 Japan; 7 Research Center for Medical Bigdata, National Institute of Informatics, Hitotsubashi 2-1-2, Chiyoda-ku, Tokyo, 101-8430 Japan

**Keywords:** Substantia nigra, Neuromelanin MRI, Segmentation, Longitudinal analysis, Generalization ability, Parkinson’s disease

## Abstract

**Purpose:**

A fully automated segmentation of substantia nigra (SN) is an essential task for the development of an explainable computer-aided diagnosis system of Parkinson’s disease (PD). Since anatomical alterations of SN are vital information in PD diagnosis, a precise segmentation model should have generalization ability against spatiotemporal changes. To satisfy these requirements, we propose a fully automated pipeline with several new techniques for a volumetric image obtained by neuromelanin magnetic resonance imaging.

**Methods:**

We develop a pipeline by integrating SN-prior probability estimation into the decision of the SN-contained region of interest. An estimated SN-prior probability is further fed into a new priority attention mechanism as a gating signal in our segmentation model. Furthermore, we introduce test-time dropout to improve a segmentation model’s accuracy and generalization ability. To evaluate the model’s generalization ability, we collected principal and external datasets with longitudinal scans of the same PD patients.

**Results:**

Our segmentation model achieved averaged Dice scores of 0.845 and 0.851 for SN hyperintense regions in the principal and external datasets, respectively. These results demonstrated the best generalization ability in our comparative evaluations. Thresholding the number of voxels in the SN hyperintense regions, we also evaluated the segmentation results in automated PD identification. The PD identification achieved the areas under the receiver operating characteristic curves of 0.755 and 0.726 by our pipeline’s output and the ground truth, respectively.

**Conclusions:**

The proposed pipeline, where we integrated SN-prior probability estimation, priority attention mechanism and test-time dropout to our segmentation model, achieved accurate SN segmentation with high generalization ability for our longitudinal data: the principal and external datasets. As demonstrated in the validation with the automated PD identification, our pipeline has the potential for improving the performance of PD diagnosis via further large-scale longitudinal analysis.

## Introduction

The patient population of Parkinson’s disease (PD) has reached 4 million nowadays in this aging society and is predicted to be 9.3 million in the coming decade [1]. PD patients typically suffer from continuous damage to the motor functions in their daily lives, which places great importance on the diagnosis and treatment in the early stage of PD. Research have revealed the progressive loss of dopaminergic neurons in the substantia nigra (SN) as one of the most commonly reported anatomic alterations related to the motor dysfunction of PD. The significant loss of a volume of SN is one of the most common findings from MRI data among PD patients [2, 3]. Therefore, automatic and precise segmentation of SN from MRI data becomes increasingly vital for further investigations of PD’s mechanisms and biomarkers toward PD diagnosis.

The neuromelanin magnetic resonance imaging (NM-MRI) highlights the dopaminergic neuron-rich regions. The NM-MRI is a more promising sequence than T1- and T2-weighted MRI for observing PD-related anatomy alterations [4]. The previous work reported that the features computed from local intensities of SN are discriminative for automatically identifying PD patients [5]. This feature computation requires a large NM-MRI dataset, where visual inspections from human experts are necessary for manually delineating the SN. To reduce the labor burden of this manual SN annotation, various semi-automated methods, intensity-thresholding, region-growing algorithms and so on, for manually cropped region of interest (ROI) have also been proposed. However, the fully automated segmentation of SN from an NM-MRI volume is still challenging due to SN’s tiny size, vague boundary, and large morphometric variability.

For the segmentation of MRI sequences, atlas-based method is one of essential approaches. To handle the anatomical variation of the small structure like SN, the multi-atlas segmentation method is proposed [6]. However, this is time-consuming since the pair-wise registration between the input and each atlas is required, even though several acceleration techniques were proposed as follows: rough alignment of a volume with predefined reference mesh [7], dynamical selection of atlases [8] and level-set methods [9, 10].

Deep-learning techniques have shown noticeable performances for multiple tasks, especially for medical image segmentation [11]. To improve SN-segmentation accuracy in 2D U-Net [11], a single atlas is introduced into ROI localization [12]. For SN segmentation of a slice image extracted from an NM-MRI volume, a two-stage method that consists of two fully convolutional networks (FCNs) has been proposed [4]. This method segments the midbrain by one U-Net at its first stage. After the normalization of an input image with the segmentation result of the midbrain, the other U-Net precisely segments SN at the second stage. However, to input an appropriate slice image clearly showing SN, an expert radiologist is required to select an input slice and normalize it manually. For a three-dimensional volume, by using dilated convolutions [13], a variant of 3D U-Net: Nigranet has been proposed [14]. However, this NigraNet also requires manual preprocessing of ROI detection and normalization. On the other hand, a 3D coarse-to-fine method with cascaded FCNs has been proposed [15], where the first 3D U-Net roughly segments SN from a downsampled MRI volume and locates an ROI for SN. The second 3D U-Net then precisely segments SN from ROI with the original resolution. Although this coarse-to-fine FCN achieved a fully automated volume-to-volume pipeline, the method requires double memory in graphic processing unit (GPU) compared with a single U-Net.

To address these problems, we propose a single-stage FCN with several novel techniques for SN segmentation from an NM-MRI volume. We summarize the main contributions of this work as follows: (1) We propose a fully automated pipeline for three-dimensional SN segmentation by introducing an automated registration as a preprocessing. The registration enables us to find ROI precisely with lower computational complexity than the cascaded FCNs. (2) We improve the accuracy and generalization ability of the segmentation models by introducing a new gated attention mechanism and the test-time dropout into 3D U-Net. In our evaluation of the generalization, we compared ours with the widely-used encoder-decoder models by using our longitudinal data. (3) The proposed pipeline’s segmentation results lead to competitive PD-identification performance with a physician.

**Fig. 1:**
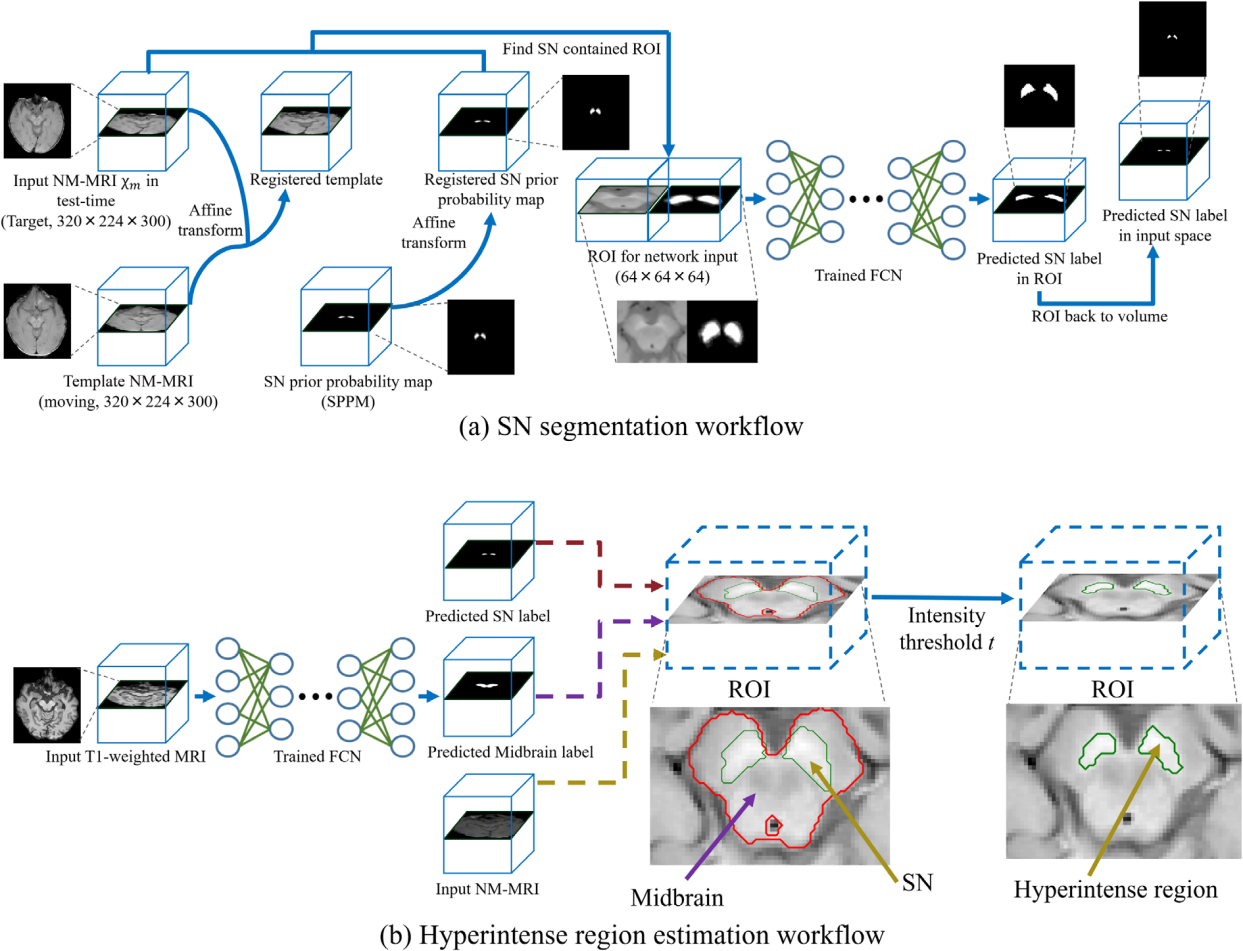
Proposed pipeline for SN segmentation (**a**) and hyperintense estimation (**b**)

## Methods

### Proposed segmentation pipeline

The proposed pipeline is summarized in Fig. 1a. The pipeline input is an NM-MRI volume, and the output is a segmented SN volume. To automatically locate the ROI fed to the trained FCN, we register the template and SN-prior probability map (SPPM) to the input NM-MRI volume by an affine transform. From these registered input and SPPM, we decide the cubic ROI centered on the voxel with the highest SN-prior probability.

#### SN-prior probability estimation

The three-dimensional SPPM estimated from the training data indicates the prior probability of SN at each voxel in the template volume, and the pair of SPPM and template needs to be generated before the FCN’s training. A random subset of the training dataset is used to accelerate the SPPM’s estimation.

In detail, we first choose a template candidate volume $\mathcal {X}_t$ from the subset of 20 NM-MRI volumes. We register $\mathcal {X}_t$ to all the other volumes in the subset by affine transformations and register the $\mathcal {X}_t$’s SN-annotation-label volume with corresponding affine matrices to obtain coarse SN-label volumes for all the other volumes. From registration results for each candidate volume, we compute an average Dice score between coarse SN-label and given annotation SN-label volumes over the subset. We repeat this process till all the volumes in the subset have been chosen as the candidate template, then the volume with the highest average Dice score is considered with a more averaged shape and selected as the final template shown in Fig. 1a.

We then register every other volume $\mathcal {X}_o$ in the subset to the template and register the $\mathcal {X}_o$’s annotation volume (binary label volume) by an affine transformation. After all the volumes have been registered to the template, we count the frequency of belonging to SN at each voxel. Each voxel value of SPPM is then defined as the normalized frequency in [0, 1].

**Fig. 2:**
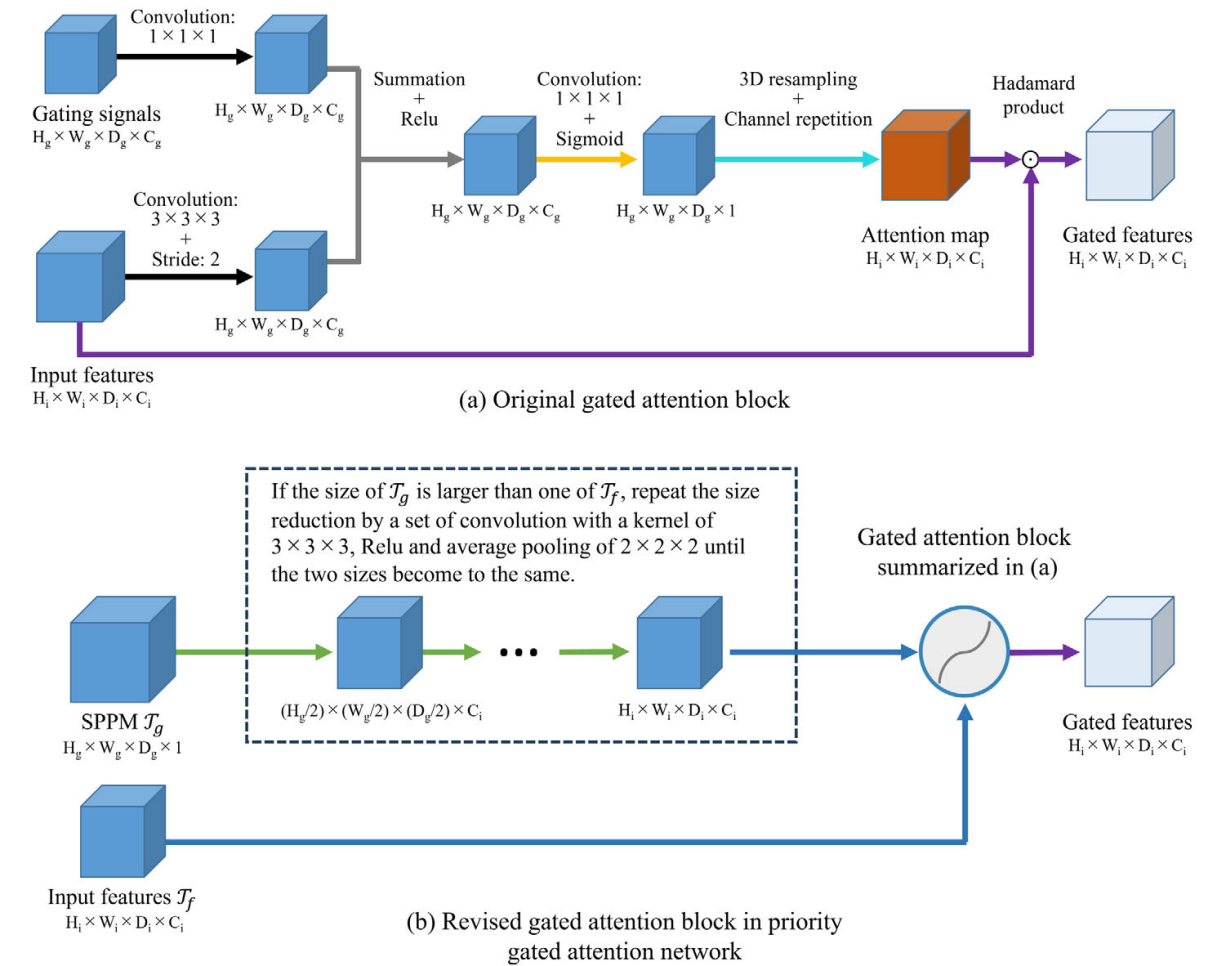
Gated attention block and SN priority-gated attention block

#### Priority attention mechanism

We propose a priority attention mechanism as an extension of the gated attention mechanism used in attention U-Net [16]. Figure 2 shows the original gated attention block and proposed. The inputs of gated attention are a feature tensor to be filtered and a gating-signal tensor to guide the computation of attention. Our attention mechanism utilizes the SPPM as a gating signal to enforce embedding SN’s spatial information [17]. Even though the original mechanism might fail to estimate accurate attention map for training with a small dataset, our approach can compute accurate one by fedding SN-prior probabilities.

**Fig. 3:**
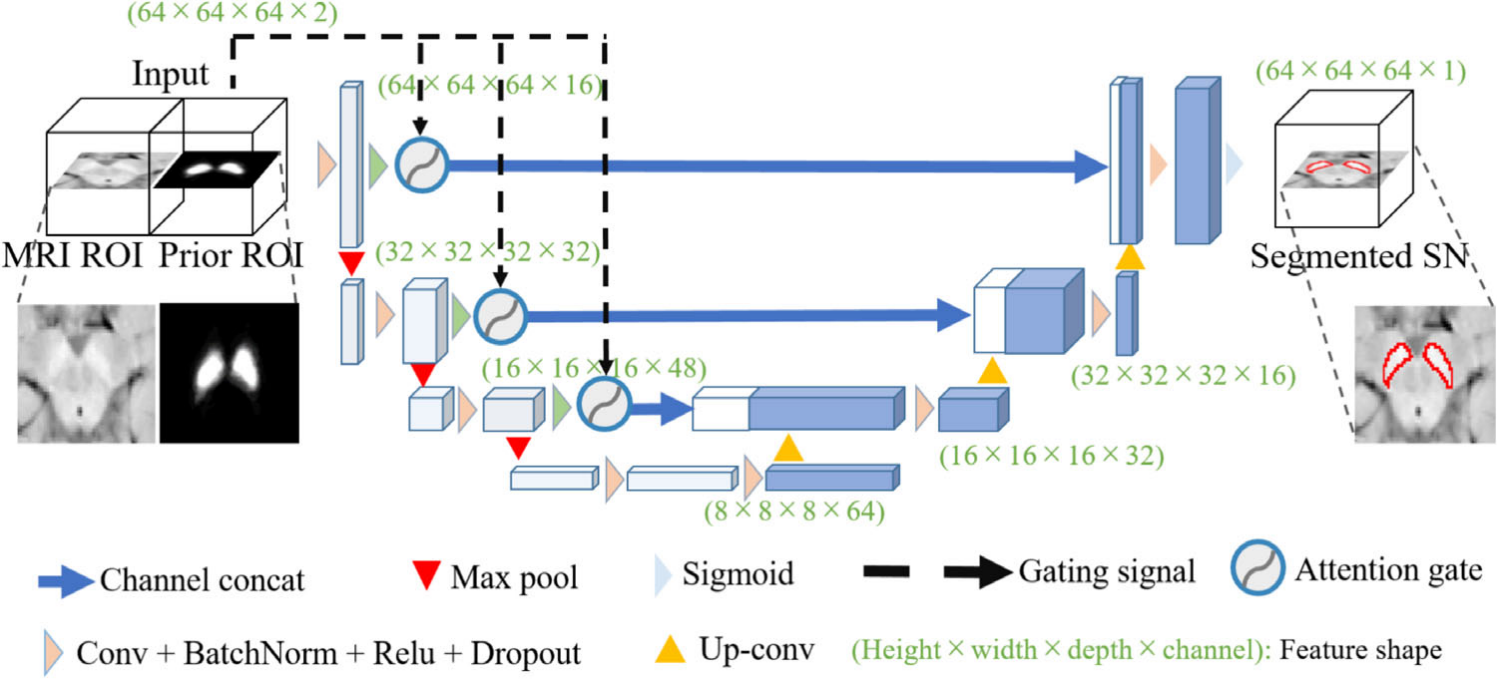
Architecture of the proposed priority-gated attention U-Net

We then propose the priority attention U-Net by using our attention mechanism. Figure 3 shows the architecture. The SPPM is convolved to the sizes of the encoder’s outputs. The outputs of the gated attention blocks are then concatenated to the decoder part in the U-Net by the skip connections between the encoder and decoder.

#### Test-time dropout

Conventionally, dropout is used to prevent overfitting to a training dataset. Recent work uses dropout in training and testing as a Bayesian approximation [18]. In a model with test-time dropout (TTD), a subset of neurons in a model are randomly selected and used for each prediction. Therefore, a model with this TTD produces several non-deterministic predictions for one input volume.

For the *m*th three-dimensional input volume in test set $\mathcal {X}_m \in [0, 1]^{H \times W \times D}$, we define a predicted probability map $\mathcal {S}=(s_{ijk}) \in [0, 1]^{H \times W \times D}$ and uncertainty map $\mathcal {U}=(u_{ijk}) \in [0, 1]^{H \times W \times D}$ as the average and standard deviation of predictions over several predictions with TTD. Setting the *l*th prediction map $\mathcal {S}_l = (s_{ijk}^{(l)})$ obtained with test-time dropout for $l=1, 2, \dots , N$, we have

1 $$\begin{aligned} s_{ijk}= &   \frac{1}{\textrm{N}}\sum _{l=1}^N {s_{i,j,k}^{(l)}}, \end{aligned}$$

2 $$\begin{aligned} u_{ijk}= &   \sqrt{\frac{1}{\textrm{N}}\sum _{i=1}^N ( s_{i,j,k}^{(l)} - s_{ijk} )^2}, \end{aligned}$$

where we set ${N}=30$.

### Hyperintense estimation

The previous study clarified the importance of hyperintense regions of SN with regard to the background structure, midbrain containing the whole SN, for PD diagnosis [4]. Therefore, automated midbrain segmentation is necessary for estimating the SN’s hyperintense region. A 2D U-Net with TTD is trained to segment the midbrain from T1-weighted MRI images. Figure 1b illustrates hyperintensity estimation, the same as the previous work [4].

The intensity threshold *t* in Fig. 1b is defined as

3 $$\begin{aligned} t = \mu + \alpha \sigma , \end{aligned}$$

where $\mu$ and $\sigma$ are the mean and standard deviation of the intensities of the midbrain regions except for the SN, respectively. We set $\alpha$ to 1.5 [4]. The hyperintense region of SN is defined as the set of voxels with larger intensities than *t*.

### PD identification

To measure the validity of the SN-segmentation results, we evaluate the accuracy of PD identification using estimated hyperintense voxels. In PD identification/diagnosis of a two-dimensional slice, a case with a hyperintense area smaller than a criteria is identified as PD (positive) [4] by a physician since the size of nigrosome 1, i.e., hyperintense region, is one of the clues for PD diagnosis. We extend this identification method for volume data. In our method, we decide the criteria for the hyperintense area of SN in the axial slice with the largest SN area in a volume. Note that the only difference from the physician’s diagnosis from NMI [4] is using automated SN segmentation results instead of a radiologist’s manual annotation.

## Experimental settings and implementation

### Longitudinal NM-MRI data

Patients diagnosed with Parkinson’s disease based on the United Kingdom PD Society Brain Bank clinical criteria and healthy subjects without any neurological diseases [19], who visited Juntendo Hospital between 2019 and 2021, were recruited. We collected T1-weighted and NM-MRI sequences of 271 cases in the first year as a principal dataset. The 130 patients are PD cases, and residual patients are healthy cases (HCs). Furthermore, after giving rasagiline mesilate to PD patients and passed one year, we collected T1-weighted and NM-MRI sequences of 54 patients, the subset of PD cases in the principal dataset, as an external dataset for our longitudinal analysis. We used a 3T scanner (MAGNETOM Prisma, Siemens Healthcare) with the following scan parameters: 600/12 ms repetition time/echo time; echo train length of 14; 2.5 mm slice thickness; 0.5 mm slice gap; 3.0 mm spacing between slices; $512 \times 359$ acquisition matrix; $220 \times 220$ mm field of view ($0.43 \times 0.43$ mm pixel size).

The manual annotations of SN were given by a board-certified radiologist with ten years of experience specializing in neuroradiology, and checked by two other experts. If the three have different opinions on the annotations, they discuss and refine them until they reach a consensus. Furthermore, to reassess our annotations, another experienced radiologist has recreated the annotations for randomly selected 20 HC and 20 PD cases in the principal dataset. We define inter-rater reliability of annotations as $r = \frac{|A_1 \odot A_2|}{|A_1|}$, where $A_1 \in \{ 0, 1\}^{H \times W \times D}$, $A_2 \in \{ 0, 1\}^{H \times W \times D}$, $\odot$, $| \cdot |$ mean our annotation, a recreated annotation, element-wise multiplication, and Frobenius norm, respectively. This measures the ratio of the intersection between two annotations. Table. 1 summarizes the main characteristics of the datasets.

We also evaluated the SN volumes before and 12 months after rasagiline administration in 54 patients with PD. We defined a volume change ratio *R* between the principal and external datasets as $R=$ (second-visit volume) / (first-visit volume). The computed means of *R* with the standard deviations over 54 PD cases are $1.10 \pm 0.52$ and $1.20 \pm 0.48$ for annotations and segmentation results, respectively, which might be affected somewhat by annotation inaccuracy. Furthermore, we defined the change ratio of MDS-UPDRS-III scores as $R_U$ = (second-visit MDS-UPDRS-III score) / (first-visit MDS-UPDRS-III score). The computed mean $R_U$ with the standard deviation is $0.79 \pm 0.35$. Moreover, we computed the Pearson correlation between *R* and $R_U$. The computed coefficients are $-3.2 \times 10^{-2}$ and $-2.0 \times 10^{-3}$, where p-values are 0.86 and 0.99, for annotations and segmentation results, respectively. The SN volume showed minimal change over the 12 months, while the MDS-UPDRS Part III scores, which reflect motor symptoms, decreased. One possible explanation for this finding is that the reduction in SN volume (suggestive loss of dopaminergic neurons) may have been suppressed by rasagiline treatment. Although the number of dopaminergic neurons in SN may have remained unchanged, the observed improvement in motor symptoms is likely attributable to the known pharmacological effect of rasagiline, namely, its ability to preserve dopamine levels through MAO-B inhibition.

**Table 1:** Clinical information of our datasets

	Principal dataset		External dataset
	HC: n=141	PD: n=130	PD: n=54
Gender (m/f)	71/70	62/68	21/33
Age	73.90 ± 6.10	64.20 ± 9.35	66.61 ± 8.55
MDS-UPDRS-III score	–	13.91 ± 7.82	10.61 ± 5.10
MMSE	–	26.33 ± 6.38	28.47 ± 1.94
annotated volume [$\textrm{mm}^3$]	219.55 ± 74.47	154.79 ± 78.10	–
segmented volume [$\textrm{mm}^3$]	239.22 ± 80.89	160.71 ± 83.30	–
*r* for whole SN	0.92 ± 0.029	0.91 ± 0.029	–
*r* for HIR in SN	0.96 ± 0.059	0.98 ± 0.035	–

HC: healthy case, PD: Parkinson’s disease patient. HIR: hyperintensity region. (mean ± standard deviation)

### Implementation

We implemented the baseline 3D U-Net and the state-of-the-art methods: NigraNet [14] and attention 3D U-Net [16] for comparison. The depth of all these investigated FCN models is kept to four. The asymmetric loss (ASL) [20] designed for tiny target segmentation is used to train all the FCNs. The hyperparameter $\beta$ of ASL is set to 1.5 according to the author’s suggestion, and the learning rate is set to 0.001 [20]. The mini-batch size is set to two. Random three-dimensional rotations are used for data augmentation. In our computations, the size of the original NM-MRI volume and ROI are $224 \times 300 \times 320$ and $64 \times 64 \times 64$ voxels, respectively.

We use the principal dataset for 10-fold cross-validation and the external dataset for evaluation of the generalization ability of the FCN models. In the cross-validation, we have training and testing sets, where we randomly separated 20 volumes of the training sets as validation sets. We trained all the FCN architectures for 1000 epochs. For each FCN, we selected the models with the highest averaged Dice score in the validation set for each FCN to compute the evaluation metrics in the testing set in each fold. As a result, we acquired 10 trained models for 10 folds for evaluations. To compute the evaluation metrics in the external dataset, we selected the model representing the averaged performance from the 10 trained models in the cross-validation.

Furthermore, we extracted the segmented SN from the results of the testing set in each fold. We computed the accuracy of PD identification over a total of 271 segmented SN. By changing the identification criteria, we plotted the receiver operating characteristic (ROC) and computed the areas under the curve (AUC) with two- and three-dimensional processing.

Moreover, for the midbrain segmentation, we randomly selected 30 HCs as training set, 20 HCs as validation set and 117 PDs as testing set from the principal dataset.

## Results

**Fig. 4:**
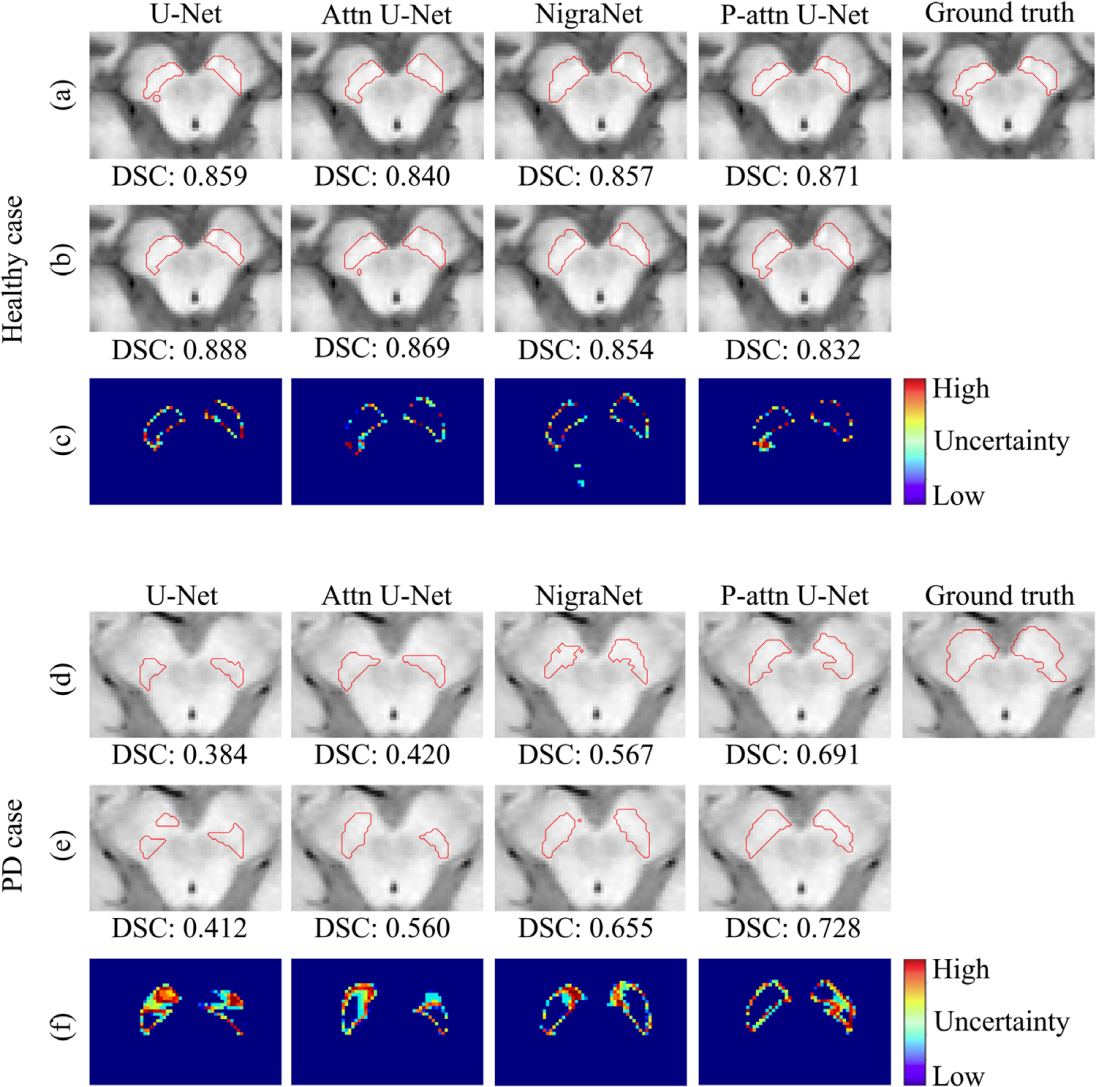
SN Segmentation and uncertainty estimation results. The top and bottom sets of three rows show the results of HC in the principal dataset and the results of PD case in the external dataset, respectively. Segmentation without TTD: **a** HC, **d** PD. Segmentation with TTD: **b** HC, **e** PD. Uncertainty estimated with TTD: **c** HC, **f** PD

We computed the Dice score, sensitivity and precision (mean±standard deviation) to quantify segmentation performance. Tables 2 and 3 show the segmentation results for models without TTD and with TTD, respectively. Figure 4 illustrates the difference among models with and without TTD, where uncertainty estimation results are also presented. Note that Fig. 4 shows the difficult cases, where a PD case has a larger SN region than non-PD case, with ambiguous boundaries of SNs for presenting the real difficulty of MRI-based PD prediction. Figure 6 presents the attention maps from the original attention U-Net and proposed network.

### Quantitative evaluations of models without TTD

Table 2 summarizes the results of the models without TTD. On the principal dataset, U-Net achieved the highest averaged Dice score (**0**.**762**) and sensitivity (**0**.**789**), and NigraNet achieved the highest precision (**0**.**777**). On the external dataset, priority-gated attention U-Net achieved the highest averaged Dice score (**0**.**758**) and precision (**0**.**776**), and U-Net achieved the highest averaged sensitivity (**0**.**756**). *Only priority-gated attention U-Net achieved almost the same segmentation accuracy in the principal and external datasets*.

### Quantitative evaluations of models with TTD

Table 3 summarizes the results of the models with TTD. On the principal dataset, U-Net with TTD, NigraNet with TTD and Attention U-Net with TTD achieved with the highest averaged Dice score (**0**.**776**), precision (**0**.**777**), and sensitivity (**0**.**814**), respectively. On the external dataset, attention U-Net with TTD achieved the highest averaged Dice score (**0**.**774**) and sensitivity (**0**.**797**). The priority-gated attention U-Net achieved the highest average precision (**0**.**778**). By applying TTD to the models, we improved the averaged Dice scores on both the principal and external datasets from the results of the models without TTD.

Additionally, we evaluate the segmentation results of TTD-introduced models for SN hyperintense regions. *Only priority-gated attention U-Net achieved almost the same segmentation accuracy in the principal and external datasets*.

**Table 2:**
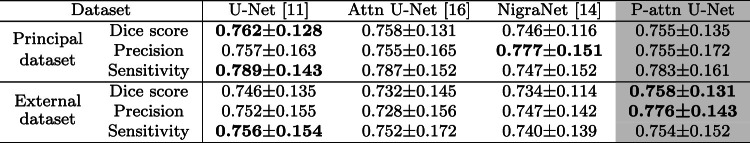
Quantitative evaluations (mean ± standard deviation) of the models without TTD in principal and external datasets

The bold values indicate the highest mean results among all methods

**Table 3:**
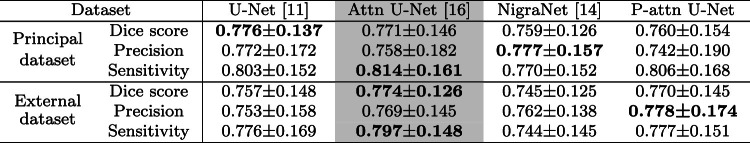
Quantitative evaluations (mean ± standard deviation) of the models with TTD in principal and external datasets

The bold values indicate the highest mean results among all methods

**Table 4:**
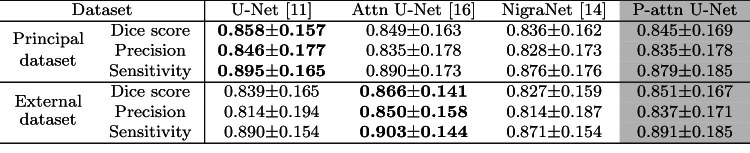
Quantitative evaluations (mean ± standard deviation) of SN hyperintense regions by the models with TTD in principal and external datasets

The bold values indicate the highest mean results among all methods

**Fig. 5:**
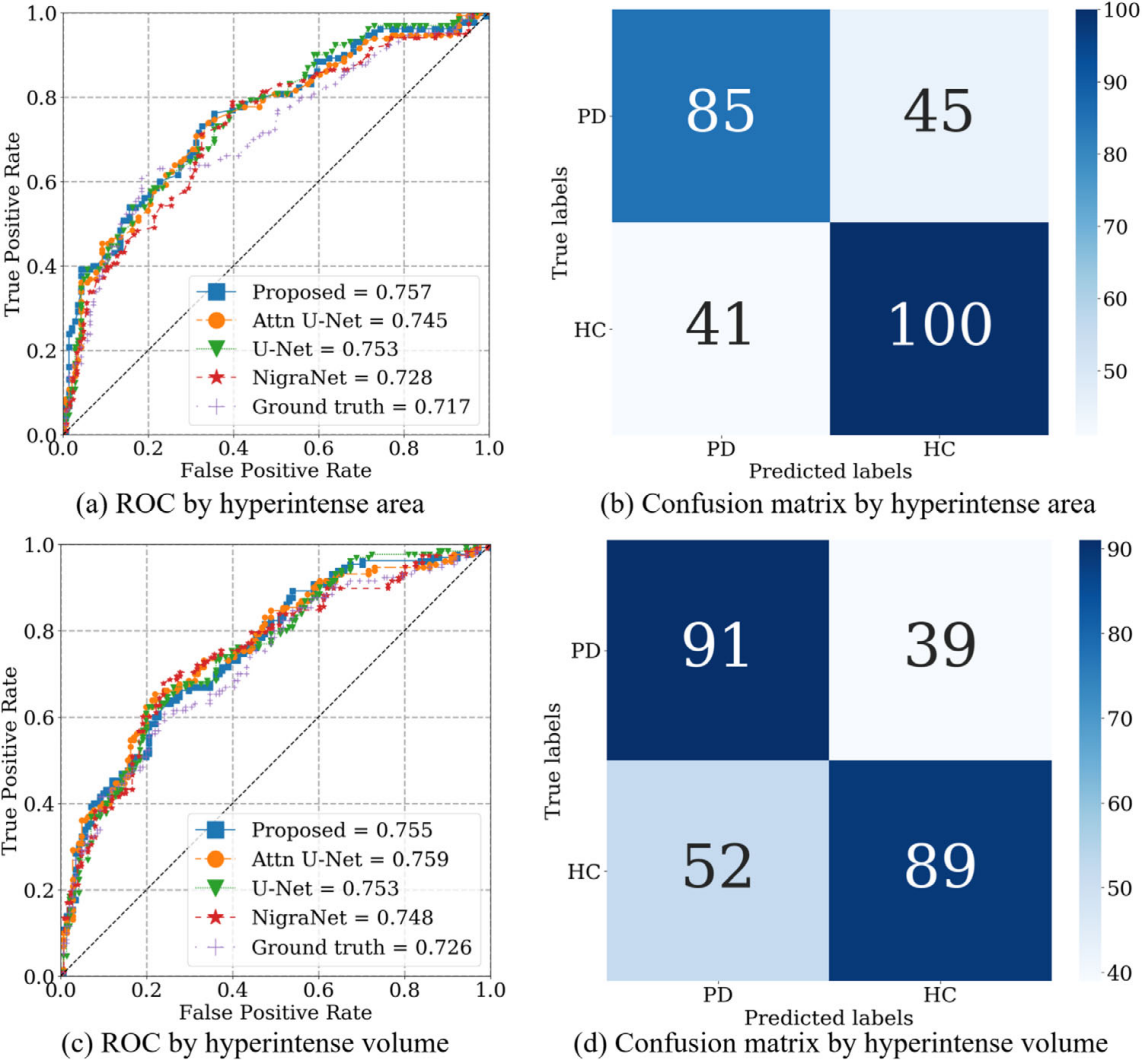
ROC curves and confusion matrices of PD identification. ROC curves by segmented regions: **a** hyperintense area, **c** hyperintense volume. Confusion matrices using segmented regions by the proposed method: **b** hyperintense area, **d** hyperintense volume

**Fig. 6:**
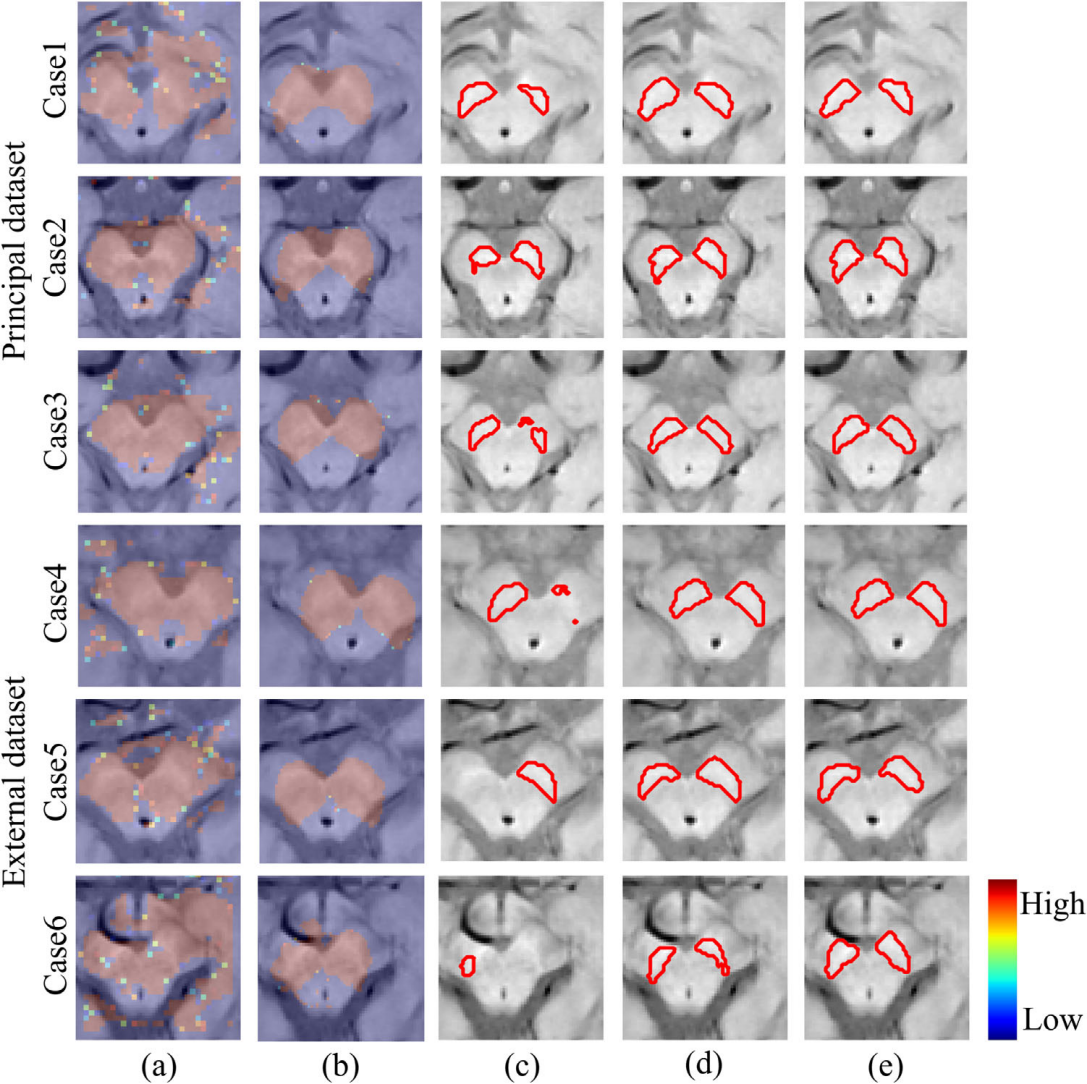
Attention maps and segmentation results. Attention maps by different networks: **a** attention U-Net, **b** proposed P-attn U-Net. Segmentation results by different networks: **c** attention U-Net, **d** proposed P-attn U-Net. Ground truth: **e**. Case1 to 3 are from the principal dataset. Case4 to 6 are from the external dataset

### PD identification

Figure 5a and c shows the ROC curves for PD identification using a hyperintense area and volume, respectively. For hyperintense estimation, we used SN regions given by the TTD-introduced models and ground truth. In Fig. 5a, *priority-gated attention U-Net with TTD (our proposed) achieved the highest AUC of* **0** *.* **757** , while the AUC for ground truth is 0.717. Even in Fig. 5c, our method achieved a higher AUC of **0**.**755** than the one of ground truth (**0**.**726**).

Figure 5b and c shows the confusion matrix of the PD-identification results for SN hyperintense estimation, where SN regions are segmented by priority-gated attention U-Net with TTD for an area and volume, respectively. In Fig. 5b, using the recommended SN hyperintense area threshold of 61.2 mm^2^ [4], we obtained an accuracy of 0.683, precision of 0.675 and sensitivity of 0.654. In Fig. 5d, using the cut-off value of 400 voxels, we obtained an accuracy of 0.664, precision of 0.636 and sensitivity of 0.700.

### Midbrain segmentation

**Fig. 7:**
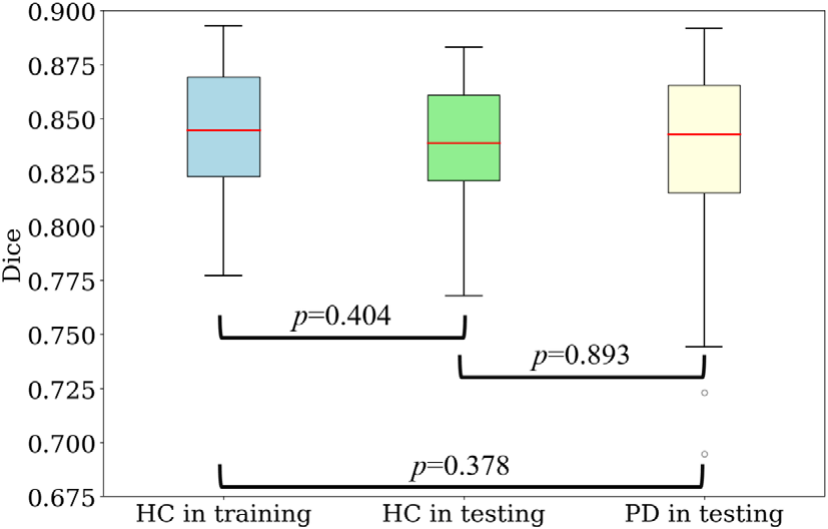
Boxplots of the Dice score for the midbrain segmentation network. The Mann–Whitney U-test is performed

Table 5 shows the midbrain-segmentation performance. In all these three sets, the segmentation achieved the average Dice score over 0.800. Figure 7 shows the boxplots of Dice score in these three groups.

### Characteristics of PD identification

**Fig. 8:**
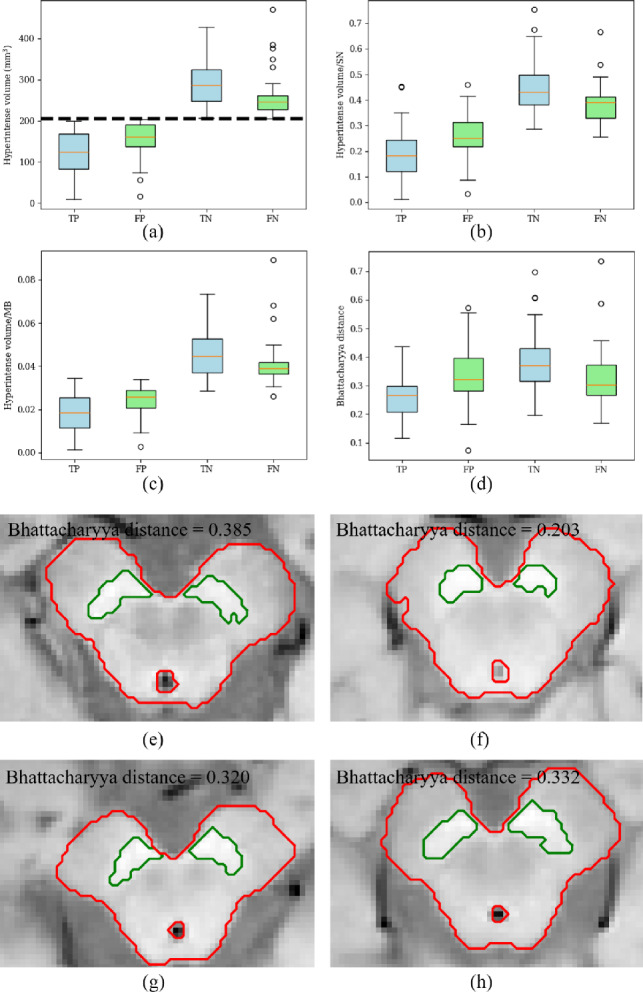
Boxplots of SN hyperintense volume and ratio. **a** SN hyperintense volume, **b** SN hyperintense volume to SN volume ratio, **c** SN hyperintense volume to midbrain volume ratio. **d** Bhattacharrya distance. **e**–**h** are examples of TN, TP, FP and FN, respectively. The dash line in **a** indicates the threshold of 204.80 $\text {mm}^3$. TP: True positive, FP: False positive, TN: True negative, FN: False negative, MB: midbrain

**Fig. 9:**
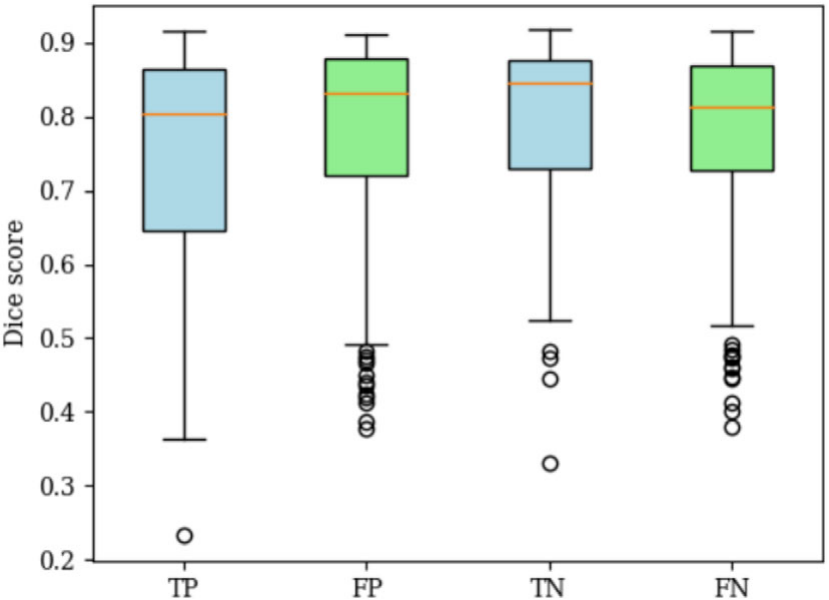
Dice score distribution in principal dataset

Figure 8 shows the boxplots of the SN hyperintense volume, SN hyperintense volume ratios and Bhattacharrya distance between the midbrain’ and SN’ intensity distributions for all the testing sets in 10 folds of the principal dataset. Figure 9 summarizes the distribution of Dice scores of SN segmentation for Fig. 8.

**Table 5:** Quantitative evaluation of midbrain segmentation in training and testing sets

	Training set	HC in testing set	PD in testing set
Dice	**0**.**845** ± **0**.**031**	0.839 ± 0.027	0.836 ± 0.038
Precision	0.806 ± 0.053	0.806 ± 0.041	**0**.**807** ± **0**.**062**
Sensitivity	**0**.**890** ± **0**.**024**	0.877 ± 0.031	0.872 ± 0.040

The bold values indicate the highest mean results among all methods

## Discussions

We evaluated the proposed and well-known FCN models with and without TTD by accuracy and generalization ability in our pipeline for the SN segmentation. In addition to the cross-validation in the principal dataset, we used the external dataset for the evaluations. Table 2 shows that the priority-gated attention U-Net achieved the highest generalization ability among the models without TTD. Since our priority-gated attention U-Net is designed to learn the input’s spatial information on different scales, our mechanisms select essential geometric features by using SPPM. Therefore, the proposed model might extract common and discriminative features in the principal and external datasets. In Fig. 6, the attention maps of the proposed network are concentrated on SN and their spatial distributions are stable across the cases from the principal to external datasets, while those of the original attention U-Net are unstable across the cases with noteworthy parts outside of midbrain. These results imply the more contribution of ours to increasing the generalization ability than the original.

Even though the original U-Net achieved the highest mean Dice score on the principal dataset, there was a gap in accuracy between principal and external datasets. Table 3 shows the lesser gap in accuracy between principal and external datasets compared with the results in Table 2. Figure 4 shows that TTD improves the segmentation results for the different models.

Furthermore, TTD enables us to estimate uncertainty in segmentation. The uncertainty maps clarified that the low confidence of the segmentation in vague boundaries and irregular shapes. Compared with HCs, the SN shapes of PD patients tend to be corrupted due to their loss of neurons. Therefore, uncertainty maps also indicate the atrophied regions related to the progression of PD. These uncertainty maps might be helpful to further analysis of SN and refinement of SN annotations. These results clarify that TTD can improve the generalization ability of the FCN model in the SN segmentation.

We also evaluated the models by using SN hyperintense estimation. Since alternations of the SN hyperintense region is a key information for diagnosing PD, the precise SN hyperintense estimation from the segmentation results is essential toward the longitudinal analysis of PD. Figure 7 shows the midbrain segmentation model achieved almost the same Dice scores between training and testing sets. Since the early-onset PD may be regarded as the transition stage from HC to PD, our model may be also helpful to the diagnosis of the early-onset PD. As shown in Table 4, the priority-gated attention U-Net achieved the highest generalization ability in segmenting SN hyperintense regions.

To evaluate the potential of the segmentation models, we also evaluated the PD-identification accuracy using all models’ SN hyperintense estimation results. As summarized in Fig. 5, the AUCs for the automated SN segmentation and ground truth are competitive. In current clinical practice for PD, cranial MRI is indispensable as it allows for excluding other Parkinsonian disorders based on their characteristic abnormalities. Our results from the ROC analysis suggest that cranial MRI alone may have potential diagnostic value in PD. These results indicate the promising potential of the proposed pipeline for the longitudinal analysis of PD.

On the other hand, in Fig. 8a, the medians of FP and FN cases are close to the threshold of 204.80 $\textrm{mm}^3$. Even in Fig. 8b and c, different measurements fail to present discriminative property. As shown in Fig. 9, there is no significant difference in segmentation accuracy among four groups. These results indicate that additional information is necessary for the PD diagnosis, except for the hyperintense volume size. Furthermore, the Bhattacharrya distance is calculated to quantify the contrast between the intensity distributions of the SN and midbrain. Figure 8d shows that PD cases, especially TP cases, have low contrast between the midbrain and SN, while many of FP and FN cases have the intermediate-level contrast between TP and TN cases. This implies that the contrast information might be useful for our future work, namely, improving automated PD identification.

Compared with the atlas-based methods and prior shape models [6, 7], our method achieves higher processing speed since ours requires only affine-transform estimation and the FCN’s segmentation. Second, ours improved generalization ability toward longitudinal analysis. To the best of our knowledge, our method is the first fully automated method tested with longitudinal datasets. Third, we evaluated the potential of our segmentation results for assisting PD diagnosis. Despite there are SN-segmentation methods [7, 9, 10, 12], few works investigated whether the segmented SN can be applied to diagnose PD. As the limitation, the proposed method requires relatively larger amount of manual annotation than the atlas-based methods [6, 8]. The second limitation is unclear robustness against different scanners and parameter settings due to the absence of a publicly available SN-annotated NMI dataset.

## Conclusions

This paper proposes the fully automated pipeline with the new encoder-decoder model for three-dimensional SN segmentation from an NM-MRI volume, and experimentally evaluated the performance of it in terms of segmentation accuracy and generalization ability. Different from the previous SN-segmentation pipeline [4, 14], the proposed pipeline avoids the manual selection of ROI and realizes the fully automated three-dimensional SN segmentation. Furthermore, we proposed the priority attention mechanism to enhance the model’s generalization ability. To further improve the model’s generalization ability, we also adopted TTD. The experimental evaluations with the longitudinal NM-MRI data demonstrated the highest generalization ability of the proposed model in the pipeline for morphological pattern changes. Moreover, we clarified that the SN hyperintense estimation from the automated SN-segmentation results is effective in PD identification. These results indicate our pipeline’s potential for the longitudinal analysis of PD.
